# Global trends in the management of cancer through obesity reduction: a bibliometric based systematic literature review

**DOI:** 10.3332/ecancer.2025.1857

**Published:** 2025-02-25

**Authors:** Muhammad Hassaan Wali, Hamza Javed, Nisar Ahmad, Ikram A Burney

**Affiliations:** 1 University College of Medicine and Dentistry, University of Lahore, Lahore 54792, Pakistan; 2 Post-Graduate Resident in Diagnostic Radiology, Ayub Teaching Hospital, Abbottabad 44020, Pakistan; 3 Sultan Qaboos University, Muscat 123, Oman; 4 Women Health Program, Sultan Qaboos Comprehensive Cancer Care and Research Center, Muscat 123, Oman; 5 Panjwani Center for Molecular Medicine and Drug Research, International Center for Chemical and Biological Sciences, Karachi University, Karachi 75270, Pakistan

**Keywords:** obesity, overweight, body mass index, body weight, cancer, neoplasm, prevention, bariatric surgery, weight loss, weight reduction

## Abstract

**Background:**

The escalating prevalence of obesity poses increased risk for public health, including an increasing incidence of cancer. The association between obesity and cancer has become an area of increasing concern and investigation. Literature on the treatment of obesity leading to a reduction in the incidence of cancer and as an adjunct to cancer-directed therapy is beginning to emerge. We conducted a bibliometric analysis to study the current trends in published literature.

**Objectives:**

The aims of the study were to explore the evolving landscape of obesity-related cancer management and identify the current areas of active research in the field.

**Methods:**

We searched the SCOPUS database on December 11, 2023, to identify the content and patterns of the literature published on the subject of ‘treatment of obesity to prevent or treat cancers’ using keywords, ‘(obesity OR overweight OR ‘Body Mass Index’ OR ‘body weight’) AND (cancer OR neoplasm) AND (prevention OR ‘bariatric surgery’ OR ‘weight loss’ OR ‘weight reduction’)’ in the title, abstract or the author-supplied keywords. After removing non-English and non-journal articles, a manual search was carried out to ensure relevance to the research question. The ‘bibliometric package’ version R 4.3.2 binaries for macOS 11 (Big Sur) and higher, signed and notarised packages, were used to extract data.

**Results:**

Over the study period, a total of 724 documents were published, 624 were subjected to manual screening and 95 were identified for analysis. An increase in the number of articles was seen from 2017 onward. ‘Bradford’s law’ was applied, and 5 core journals published 33/95 (34.7%) of all articles and received 1,808/4,399 citations (41.1%). The vast majority of articles, reported on the use of bariatric surgery for weight reduction as a method for cancer prevention and as an adjunct to cancer-directed treatment.

**Conclusion:**

The treatment of obesity seems to be emerging as a strategy for the prevention and treatment of cancer. The field is relatively new, publications have begun to emerge in the last 10 years, and there is a growing interest in bariatric surgery as a method to prevent obesity-related cancers.

## Introduction

The global burden of obesity has reached epidemic proportions in the last 50 years. The escalating prevalence of obesity poses increased risk for public health, including non-communicable diseases, such as cancer [1]. Therefore, the association between obesity and cancer has become an area of increasing concern, and investigations. Thirteen different obesity related cancers that make up 4%–8% of all cancers have been attributed to obesity [1,2]. A meta-analysis of 203 studies involving 6.3 million cancer patients showed that obesity was associated with a 14% increased risk of overall mortality and a 17% increased risk of cancer-specific mortality [2]. However, the impact of obesity on outcomes may vary on the specific type of cancer [3]. For instance, patients with obesity and renal cell carcinoma, endometrial cancer, lung cancer or melanoma have better survival than patients without obesity [3].

The link between obesity and cancer is complex, involving biological mechanisms, hormonal disruptions and inflammatory processes [4]. Obesity triggers systemic alterations, including changes in insulin-like growth factor-1, leptin, adiponectin, steroid hormones and cytokines creating an environment conducive to the initiation and progression of cancer [5]. Additional mechanisms through which obesity may impact cancer risk include compromised immunity, shifts in the mechanical properties of adipose cells, dysregulation of gut microbiota and modification of the tumour microenvironment [6]. Consequently, elucidating the intricate biological mechanisms linking obesity to cancer assumes importance in the formulation of effective strategies for cancer prevention and treatment [6].

As the epidemiological evidence linking obesity to cancer, and the effect of obesity on cancer outcomes continues to accumulate [7–10], the data to study the potential impact of weight loss interventions on cancer incidence and outcomes have begun to emerge [11–13]. A meta-analysis was conducted to assess the effect of weight loss interventions on cancer risk reduction and associated mortality and reported low-quality evidence of incidence of new cancers (103 events; RR 0.92, 95% confidence interval 0.63–1.36), or weight reduction on cancer mortality (eight trials, 34 events; RR 0.58, 95% confidence interval 0.30–1.11) [11]. However, the Iowa women health study reported a significant cancer risk reduction in patients undergoing intentional weight loss (RR = 0.89, 95% CI 0.79–1.00) [12]. A significant reduction in cancer risk was also reported in post-menopausal women undergoing intentional weight loss, especially the endometrial and colorectal cancer risk [14].

Lifestyle modifications have long been suggested to reduce the risk of cancer. For example, calorie restriction, intermittent fasting, ketogenic diets and pharmacological agents such as metformin have been studied to enhance the effects of adjuvant chemotherapy and radiotherapy [15]. Over the last two decades, bariatric surgery has been studied for the prevention of obesity-related cancers and has shown promising results [16–18]. Different weight reduction strategies pose different challenges. Longitudinal analysis of obesity treatment and intervention research reveals that dietary adjustments and lifestyle modifications, when employed alone, yield only marginal significance in causing weight reduction. Whereas sustained weight loss over time presents a formidable challenge [19, 20], bariatric surgery demonstrates encouraging outcomes in the short term. However, enduring maintenance of weight loss emerges as a concern, particularly considering the prevalence of associated deficiencies in essential vitamins and minerals over prolonged periods [21, 22].

As the association between treatment of obesity and cancer outcomes continues to evolve, we conducted a bibliometric analysis to study the pattern of published literature on the subject. The study had two primary objectives: to explore the evolving landscape of obesity-related cancer management, and to identify the current areas of active research within the field. The results will provide insights to researchers and clinical practitioners engaged in the management of cancer by treating obesity.

## Methodology

**Figure 1.**
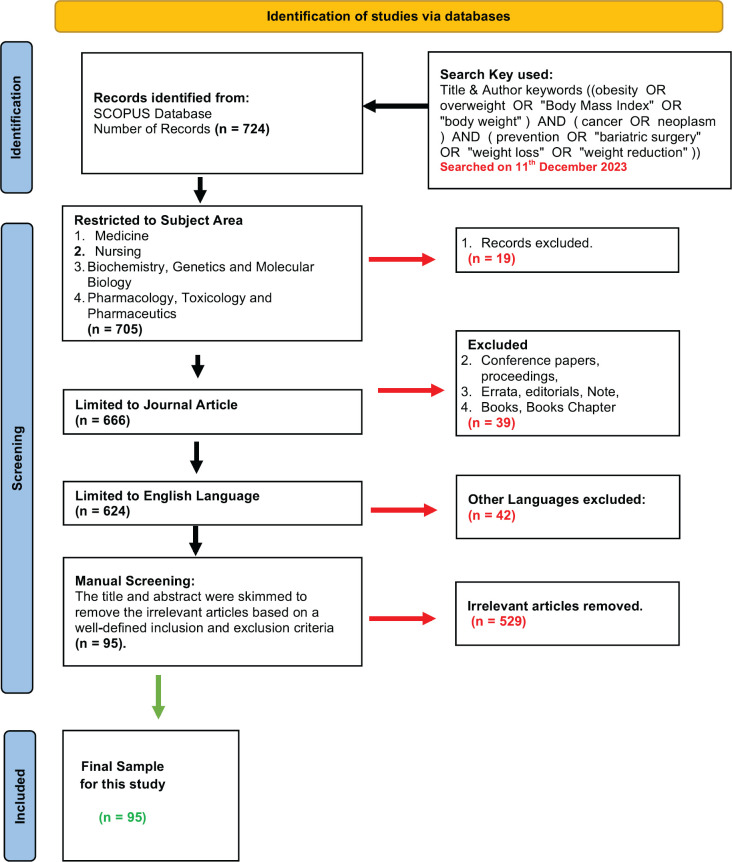
Article selection flow chart (PRISMA).

A comprehensive search strategy, using the SCOPUS database was conducted on December 11, 2023, to identify the content and patterns of the literature published on the subject of ‘treatment of obesity to prevent or treat cancers following the PRISMA protocols (Figure 1). We searched keywords, ‘(obesity OR overweight OR ‘Body Mass Index’ OR ‘body weight’) AND (cancer OR neoplasm) AND ( prevention OR ‘bariatric surgery’ OR ‘weight loss’ OR ‘weight reduction’)’ in the title, abstract or the author-supplied keywords. A total of 724 articles were identified in the initial search. Journal articles published in English language between 2003 and 2023 within subject areas, medicine, nursing, biochemistry, genetics and molecular biology, pharmacology and toxicology and pharmaceutics were included. Conference papers, proceedings, errata, editorials, notes, books or book chapters, as well as studies in languages other than English were excluded. A total of 624 articles were subjected to manual screening. Two authors reviewed the title and abstracts, and in some cases the methods section of the full paper, to identify papers which described outcomes of cancer in relation to treatment of obesity. Wherever, there was a dispute between two investigators, a third author reviewed the manuscript independently and the paper was included or excluded by consensus. A total of 95 articles were identified. Full text of these articles was reviewed to ensure relevance to the research question.

Results are based on these 95 articles. The temporal trend of the data (number of publications or citations) was plotted over time and subjected to co-word analysis, Bradford's law and Lotka's law. The ‘bibliometric package’ version R 4.3.2 binaries for macOS 11 (Big Sur) and higher, signed and notarised packages was used to extract data. Artificial intelligence assisted technologies have been used to improve the clarity of the manuscript and improve the sentence structure and understanding.

## Results

Over the study period 2003 – 2023, a total of 95 documents (55 original articles and 40 reviews) were identified as relevant. The average age of the documents was 5.8 years and they received 46.3 citations each. Each document had 6.8 co-authors and 17.9% of publications involved authors from different countries.

**Figure 2.**
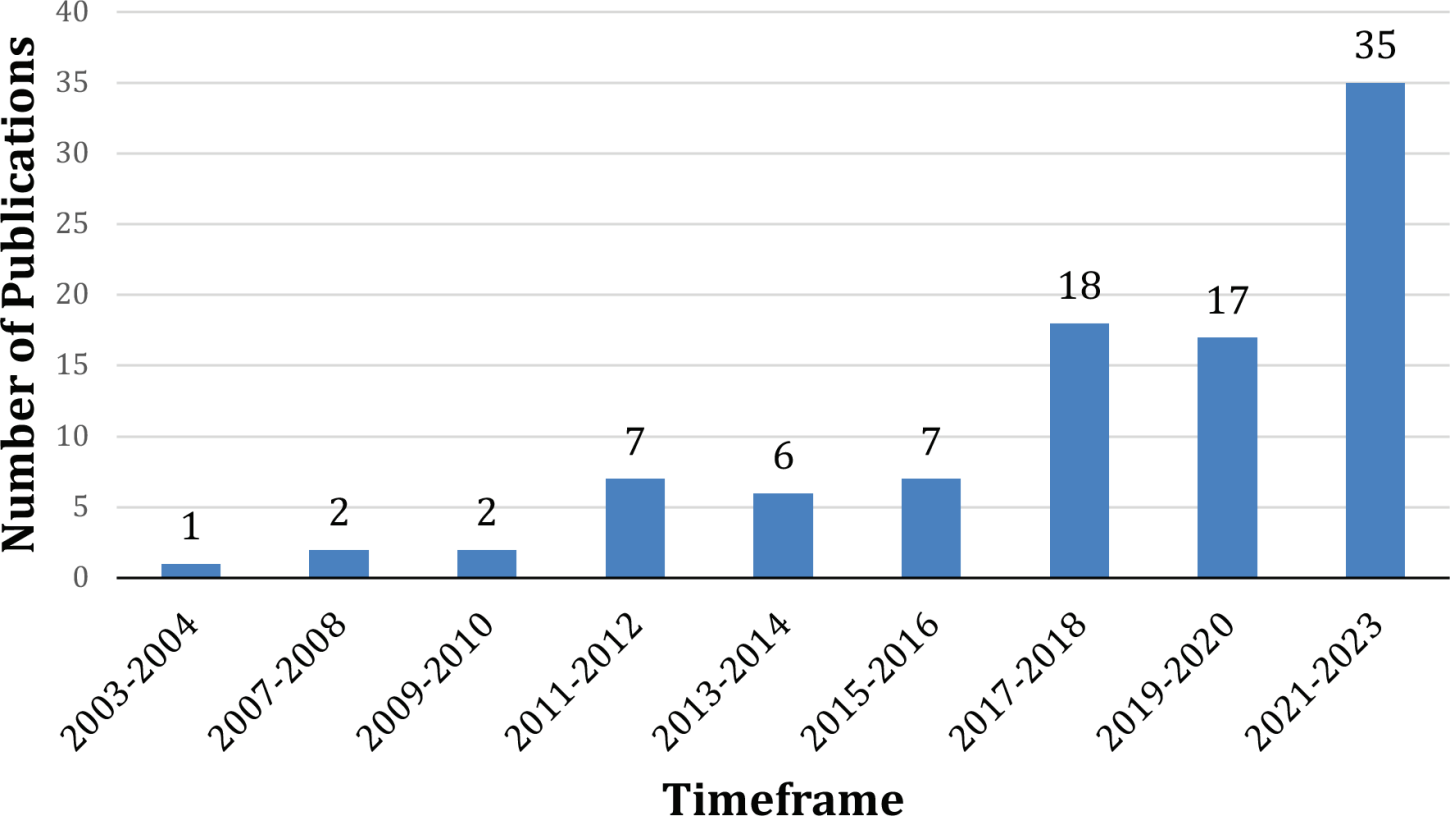
Publication trend from 2003-2023.

An increase in number of publications was observed in 2011, and since 2017, there has been a sustained increase as shown in Figure 2.

The vast majority of papers were published in journals, ‘Obesity Surgery’, ‘Gynecologic Oncology’ and ‘Cancer’. (Figure 3A). ‘CA Cancer Journal for Clinicians’ received the highest number of citations, followed by ‘Obesity Surgery’ and ‘Surgery for Obesity and Related Diseases’ (Figure 3B). Ever since 2011, journals ‘Obesity Surgery’, ‘Surgery for obesity and related diseases’ and ‘Gynecologic oncology’ published increasing number of articles (Figure 3C). According to Bradford’s law, five core journals published 33 papers, receiving 1808 citations (Figure 3D).

Figure 4A shows that 4 authors published 4 or more articles as the first author. Figure 4B shows the productivity of authors over the course of time. The vast majority of published articles emerged from the University of Manchester NHS Foundation Trust, followed by the University of California and the University of Gothenburg (Figure 4C).

**Figure 5.**
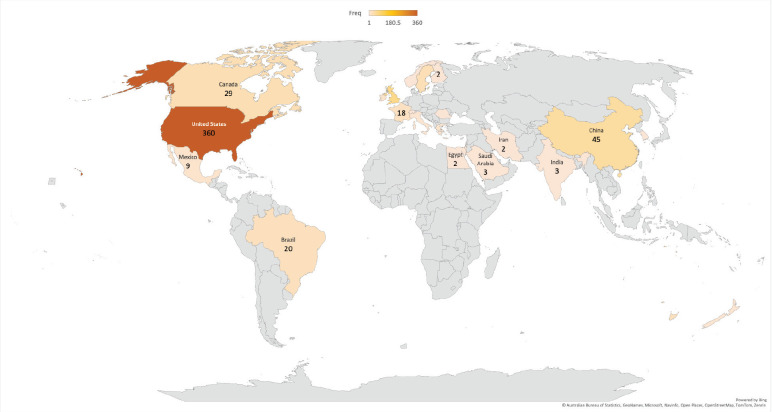
Geographical scope of the authors contributing to the research.

Insofar as authors are concerned, the vast majority belonged to either the USA, the UK or China, followed by Canada and Sweden (Figure 5). The United States leads with 360 authors, followed by the United Kingdom (65) and China (45).

The word cloud shows the most frequent terms used in the analysed text related to obesity related to cancer and weight loss, with font size indicating the word’s prominence. The largest terms, ‘obesity,’ ‘female’ and ‘bariatric surgery’ emphasise the central focus on the relationship between treatments of obese female cancer patients using bariatric surgery (Figure 6A).

Thematic analysis revealed a network of interconnected themes. At the center lies ‘obesity and weight loss,’ encompassing various strategies, interventions and outcomes related to weight management. This central theme connects to other themes such as ‘endometrial cancer’ and ‘breast cancer’, ‘metabolic surgery’ and ‘gastric bypass surgery’ ‘physical activity’ and ‘exercise’ and ‘cancer prevention’ and lifestyle guidelines’. The presence of comorbidities underscores the importance of managing co-occurring health conditions like diabetes alongside weight management (Figure 6B).

## Discussion

The bibliometric analysis spanning publications from two decades highlights the significant and evolving scholarly attention on treatment of obesity for the management of cancer, across the continuum including prevention and treatment. This analysis highlights the recent increase in number of publications, especially since 2017. Journals, ‘Obesity Surgery,’ ‘Gynecologic Oncology’ and ‘Cancer’ have steered the discourse on the subject. The universities of Manchester, California and Gothenburg are spearheading the research. Major weight management strategies include lifestyle modifications and bariatric surgery. Bariatric surgery as a method to prevent cancer or used as an adjunct to systemic treatment occupies a central place. Out of the 60 most recent publications, 41 addressed bariatric surgery.

Over the last 50 years, there has been a significant increase in the rates of obesity globally, except for parts of sub-Saharan Africa, and some parts of Asia (Indonesia, Singapore, Sri Lanka and so on). [23, 24]. It has been estimated that around 15%–20% of the world’s adult population is obese. This is in addition to around 40% of population classified as overweight (BMI > 25, but less than 30 kg/m^2^). The high prevalence rates of obesity are most evident in the developed countries, where around 40%–45% of adults are obese, and the early industrialised countries, such as some of the middle eastern countries, where the prevalence rates of obesity reach up to 30% of adult population [25]. Childhood and adolescent obesity are also significant; up to 1/3^rd^ of children in the US, and 1/5^th^ in countries like Oman are obese, and the incidence continues to increase [24]. At the same time, the prevalence rates for generalised obesity continue to rise in children and adults, with peak obesity rates occurring in the fifth–sixth decades of life. Women may have equal or greater obesity rates than men [25].

Co-incident with rise in obesity, the incidence rates of what are known as ‘obesity-related cancers’, such as endometrial cancer, colon cancer and post-menopausal breast cancer also increased steadily. For example, the incidence rates of endometrial cancer began to rise in the late 20^th^ century, and today nearly 60%–80% of endometrial cancer in the developed world are associated with obesity [26]. Nearly 70% of patients with colon cancer are obese [26]. The rise in incidence of colon cancer also coincides with westernisation of diet and sedentary lifestyle, and the incidence continues to increase, even in the younger age group [24]. The incidence rates of post-menopausal breast cancer started to increase especially in high income countries, which are known to have increased obesity [24].

The etiology of obesity is complex [23]. Traditionally, obesity was considered to occur because of excess energy resulting from food consumed and energy expended. Widespread availability of energy-dense drinks and fast foods together with a sedentary lifestyle have certainly contributed to that direction. However, other factors, such as family history and family environment play a part. For example, a child with one obese parent has a three-time risk of becoming obese, while if both parents are obese, the risk goes up 10-fold. Furthermore, healthy lifestyle of mothers was shown to be associated with a reduced risk of their children developing obesity as adults. Genetic factors also play a key role in the development of obesity. For example, genome-wide association studies identified more than 400 genes associated with diabetes mellitus, although, only a small proportion of these predict obesity. In addition to energy imbalance, family dynamics and genetic factors, intrinsic factors, such as the gut microbiome have been implicated in the development of obesity. The human body contains around 3.8 × 10^13^ microorganisms, of which more than 50% are bacteria. However, obesity occurs in people with an altered gut microenvironment with diverse viral species.

Whatever, the etiology of obesity may be, scientific rationale for connection between obesity and cancer is well described [4, 5, 27]. Adipose tissue functions like an endocrine organ. Adipose tissue secretes adipokines, mainly leptin, which induces upregulation of transcription factors through activation of PI3K, MAPK and STAT3 pathways. Also, pro-inflammatory cytokines, such as TNF-α, IL-2 and IL-10, secreted by the adipose tissue, lead to the activation of NF-ĸB. In addition, adipose tissue produces steroid hormones which lead to the activation of insulin and insulin-like growth factor 1, which in turn leads to the release of leptin and pro-inflammatory cytokines. An increase in adipokines, pro-inflammatory cytokines, steroid hormones and the resultant persistent hyperinsulinemia leads to upregulation of transcription factors. As a result, the risk of cancer progression or its recurrence is higher amongst obese individuals.

Obesity is defined as a BMI of more than 30 kg/m^2^. Obesity could be further sub-classified as Class I (BMI – 30–35), Class II (BMI 35–40) and Class III or morbid obesity (BMI >40 kg/m^2^). Classification of obesity is required not only for risk stratification, but also for implementation of weight management strategies and prevention of obesity-related co-morbidities and complications. Almost 1/5^th^ of all obese people have class III or morbid obesity. For example, US study showed an obesity-prevalence rate of 42%, and out of the entire study population, 9% had class III or morbid obesity. Bariatric surgery is indicated to reduce cardiovascular mortality in patients with Class III obesity, and in selected patients with Class II toxicity, who have multiple co-morbid illnesses [28].

The trend towards bariatric surgery for the management of cancer is not just a narrative of increased research interest but reflects a paradigm shift in how the medical community views the intersection of obesity management and cancer prevention [29]. Most of the publications emerging after 2017 highlight the recognition of metabolic surgery as an emerging frontier in the fight against obesity-related cancers. This observation coincides with increasing evidence of significant reductions in cancer incidence among individuals undergoing bariatric surgery [30]. The authors studied the incidence of numerous cancer types following either vertical sleeve gastrectomy or Roux-en-Y gastric bypass and compared to bariatric surgery eligible patients who did not undergo surgery. Individuals undergoing Roux-en-Y gastric bypass developed fewer colorectal cancers, and patients undergoing vertical sleeve gastrectomy developed fewer lung cancers compared to patients without any surgical intervention (odds ratio 0.47–0.42 respectively).

Bariatric surgery has been linked with notable declines in the incidence of several obesity-related cancers such as the post-menopausal breast cancer, prostate cancer and endometrial cancers [31, 32]. Database of the New York Statewide Planning and Research Cooperative System was reviewed to study female-specific cancer incidence among more than 55,000 obese women who did, and around 250,000 obese women who did not undergo bariatric surgery. Patients who underwent the surgery were significantly less likely to develop breast, ovarian or endometrial cancer (hazard ratio 0.78 995% CI 0.51–0.87). However, the relationship of metabolic surgery is controversial in relation to colorectal cancer. Most studies report a risk reduction, while a few also mention a potential increased risk of colorectal cancer incidence after bariatric surgery [33–36]. The nationwide cohort study of French individuals suggested that patients with obesity share the same risk of colorectal cancer as the general population following bariatric surgery, whereas for obese patients who do not undergo the surgery, the risk is 34% higher than the general population [34]. An English population-based cohort study revealed that the risk of colo-rectal cancer was high in obese individuals, a prior obesity surgery was not associated with an increased risk, however, due to limited follow up, form conclusions could not be drawn [35]. On the other hand, a multicountry Nordic cohort study revealed that in a cohort of about half-a-million obese individuals, 10% patients who underwent bariatric surgery had an increased risk of colon cancer (HR 1.55 95% CI 1.04–2.31), 10–14 years after bariatric surgery [36].

Prior to the widespread adoption of bariatric surgery, lifestyle modifications, such as dietary changes, enhanced physical activity and pharmacotherapy were the cornerstone of obesity management, showing modest effects in reducing the risk of obesity-related cancers [37, 38]. These non-surgical strategies are integral to a holistic approach in obesity management, offering viable options for individuals seeking alternative or complementary methods to surgical interventions for weight loss and cancer risk mitigation [39, 40]. However, these conventional methods are fraught with challenges, such as, non-compliance, patient dropout, weight regain and inadequate weight loss [41]. Bariatric surgery seems to provide a pivotal breakthrough, surmounting these issues by delivering lasting weight loss, thereby markedly improving obesity and related cancer risk management [42, 43]. The interconnected themes reveal the comprehensive picture of the field's current landscape and its rapid evolution. The significant focus on bariatric surgery emphasises its growing importance in reducing obesity-related cancer risks and marking a crucial area for future research and clinical practice.

There are several limitations of the study. First, only one database SCOPUS was used for literature review. However, SCOPUS is the largest database and includes more than 23,000 journals. Furthermore, all the core journals included in this review are also included in the other databases, such as PubMed. It is plausible to think that the broad landscape of the field ‘treatment of obesity for prevention and treatment of cancers was captured using the SCOPUS database. Second, the sample size was relatively small. However, the analysis conforms to Lotka's Law, showcasing a limited number of authors who significantly shaped the research landscape, especially on bariatric surgery and cancer. Applying Lotka’s law, we were also able to demonstrate the continuous influx of new authors in the field highlighting the evolving nature of research within the field.

## Conclusion

In conclusion, treatment of obesity seems to be emerging as a strategy for prevention and treatment of cancer. The field is relatively new, publications have begun to emerge in the last 10 years or so, and there is a growing interest in bariatric surgery as a method to prevent obesity-related cancers. However, the relationship is not linear, and there is a hint that the incidence of some cancers may not decrease despite the surgery.

## Conflicts of interests

None.

## Funding

Nil.

## Figures and Tables

**Figure 3A.**
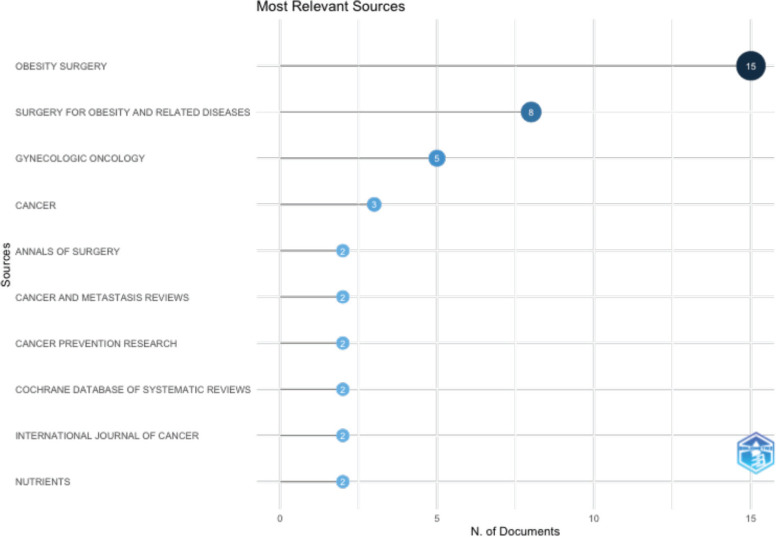
Top 10 most productive journals with their total number of publications.

**Figure 3B.**
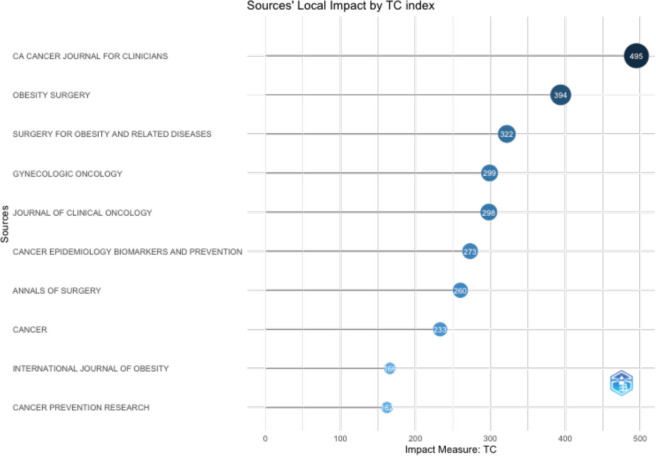
Top 10 most cited journals with their impact measures.

**Figure 3C.**
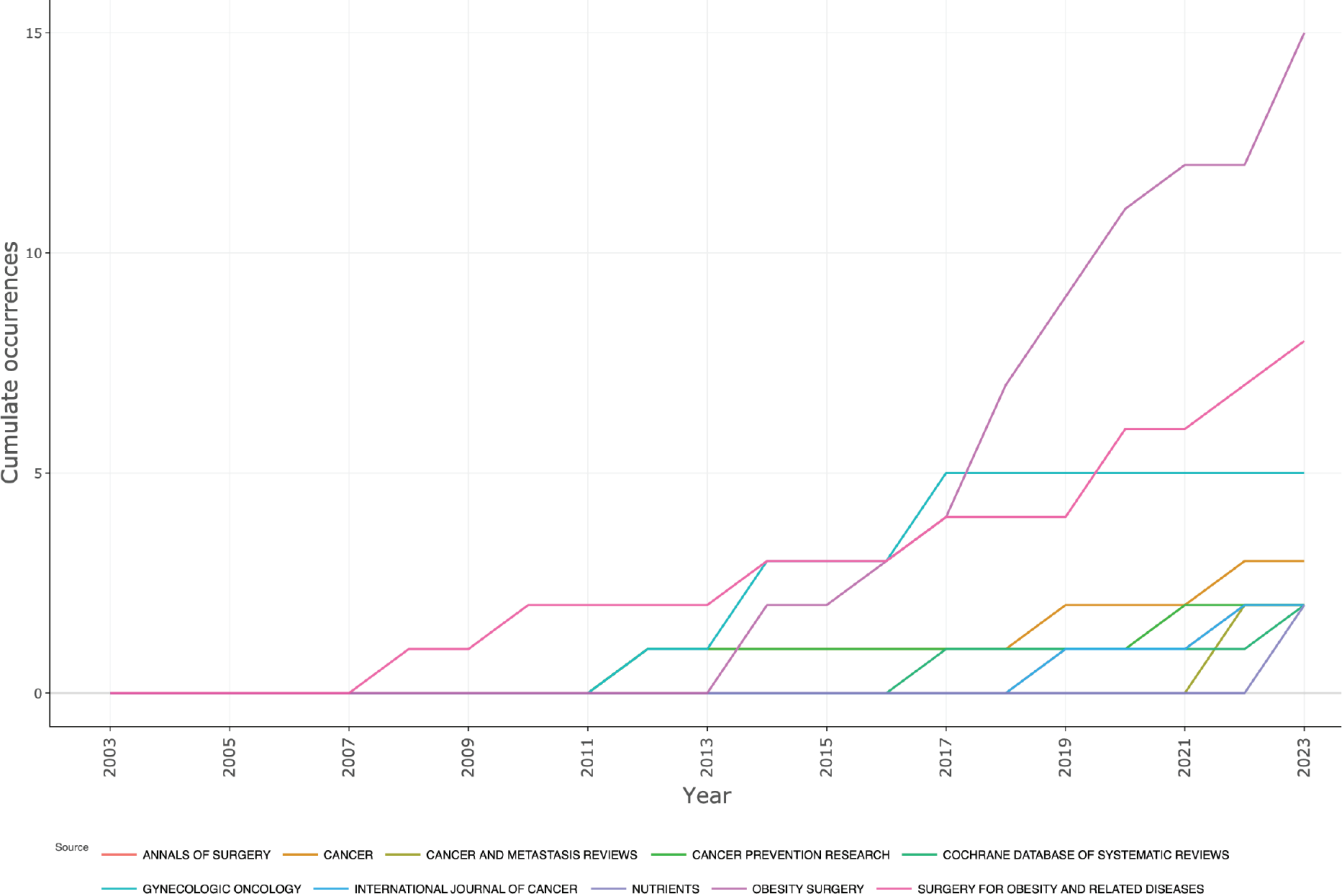
Publication trend over time in the top journals.

**Figure 3D.**
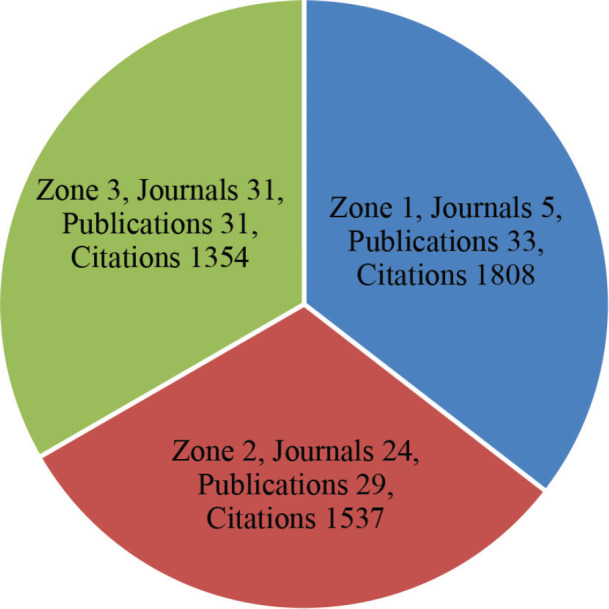
Distribution of core journals according to Bradford’s Law.

**Figure 4A.**
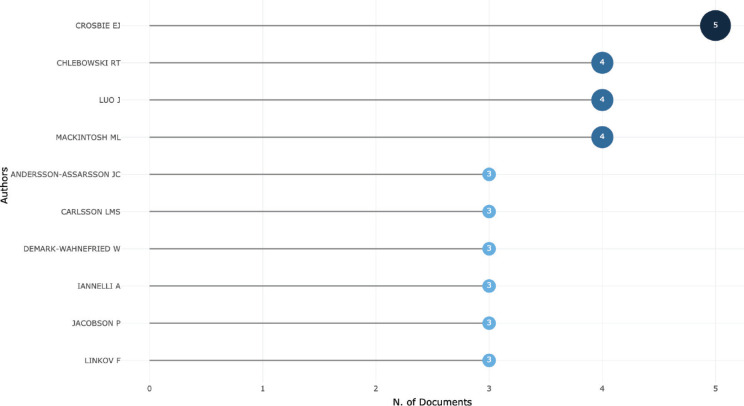
Top 10 most productive authors and the number of documents they authored.

**Figure 4B.**
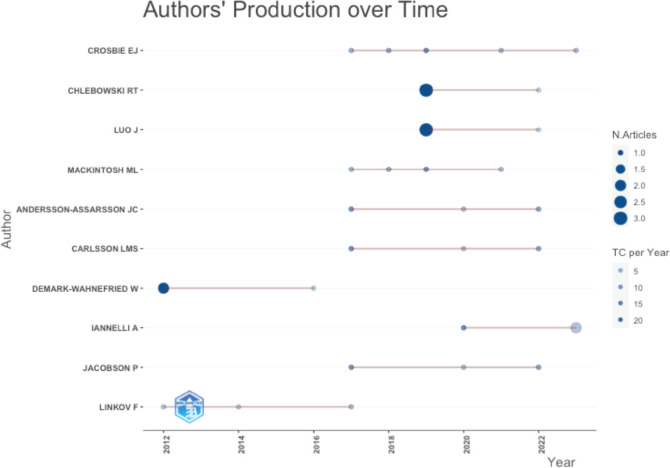
Authors' production over time as per Lotka’s law.

**Figure 4C.**
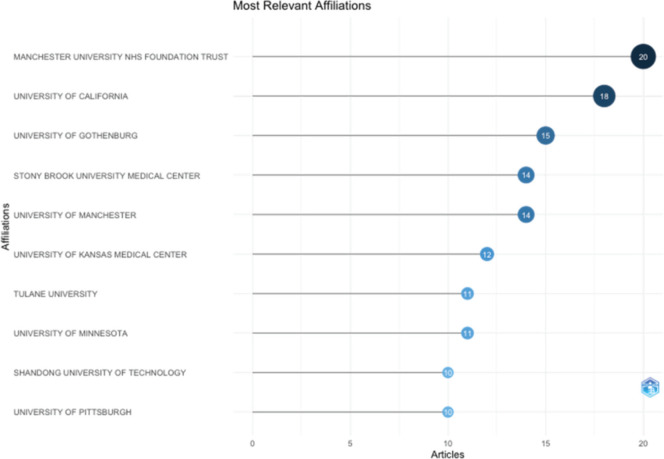
List of most common affiliated institutions of the authors.

**Figure 6A.**
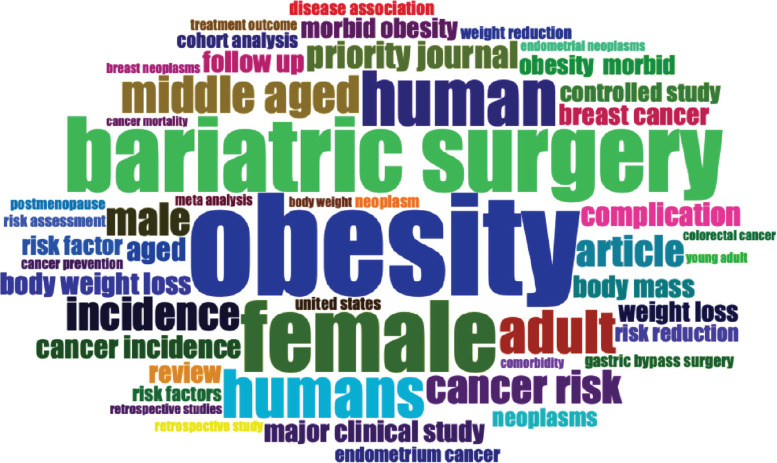
Word cloud of the keywords.

**Figure 6B.**
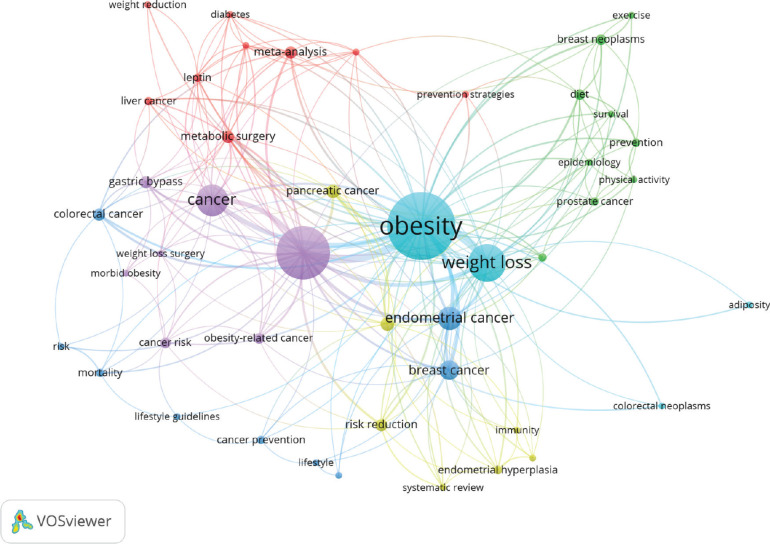
Thematic analysis of the co-occurring health conditions.
